# Coinfection With Lophomonas spp. and Streptococcus pyogenes Presenting as Atypical Pneumonia in a Patient With Poorly Controlled Type 1 Diabetes Mellitus: A Case Report From Mexico

**DOI:** 10.7759/cureus.96608

**Published:** 2025-11-11

**Authors:** Angélica Nohemí Díaz Castaño, Daniela Karina Carrillo Llanas, Rebeca Sinaí Fuentes Velázquez

**Affiliations:** 1 Infectious Diseases, Universidad Autónoma de Coahuila, Saltillo, MEX; 2 Faculty of Medicine, Universidad Autónoma de Coahuila, Saltillo Campus, Saltillo, MEX

**Keywords:** coinfection, lophomonas spp, pneumonia, pulmonary lophomoniasis, streptococcus pyogenes, type 1 diabetes mellitus (t1d)

## Abstract

Pulmonary lophomoniasis is a rare protozoal infection caused by *Lophomonas* spp., anaerobic, flagellated organisms that inhabit the gastrointestinal tract of cockroaches. *Lophomonas blattarum* is considered a public health concern due to its involvement in human bronchopulmonary infections. In contrast, *Streptococcus pyogenes*, a Gram-positive bacterium commonly associated with pharyngitis and invasive infections, plays an unusual role in pneumonia. To our knowledge, this represents the first documented case of coinfection involving *Lophomonas* spp. and *S. pyogenes*, rendering this case atypical.

We present the case of a 28-year-old female patient with type 1 diabetes mellitus and persistent poor glycemic control, who developed pneumonia concomitant with *Lophomonas* spp. and *S. pyogenes* infection and was admitted to La Concepción Hospital in Saltillo, Coahuila, Mexico. Initial chest computed tomography (CT) revealed pleural effusion and pulmonary consolidations, prompting further microbiological and immunological investigations. The presence of *Lophomonas* spp. was confirmed through bronchoalveolar lavage, which led to the initiation of metronidazole therapy. Despite initial symptom resolution, the patient experienced recurrent symptoms, and a polymerase chain reaction (PCR) panel identified *S. pyogenes*, necessitating adjustment of antibiotic therapy. This case highlights the diagnostic challenges of pulmonary lophomoniasis and underscores the importance of considering potential bacterial coinfections, particularly in immunocompromised patients such as those with poorly controlled type 1 diabetes mellitus.

## Introduction

*Lophomonas* spp. is a multiflagellated anaerobic protozoan that inhabits the digestive tract of arthropods such as *Periplaneta americana*, *Blatta orientalis*, and *Blattella germanica* [1]. The genus *Lophomonas* includes *L. striata* and* L. blattarum*, with the latter recognized as the primary etiologic agent of human lophomoniasis [2].

Lophomoniasis is a rare infection predominantly affecting the bronchopulmonary system (97%), followed by involvement of the paranasal sinuses (2%) and urinary tract (1%) [3]. Transmission is believed to occur primarily through inhalation of cysts present in cockroach feces, with airborne spread being the only confirmed route to date [4].

*S. pyogenes, *also known as Lancefield group A beta-hemolytic streptococcus, is a Gram-positive bacterium associated with a wide spectrum of clinical manifestations, ranging from mild localized infections to severe invasive diseases. Pneumonia caused by *S. pyogenes* is uncommon and represents an atypical clinical presentation [5]. Transmission occurs via respiratory droplets, direct contact with nasal secretions, or through contaminated fomites [6].

Coinfection with *Lophomonas* spp. and *S. pyogenes *is rare but clinically significant due to its implications for diagnosis and management. Both pathogens have been most frequently reported in younger populations. While immunocompromised individuals are at increased risk for such infections, cases have also been described in immunocompetent patients [2,7]. This uncommon association between *Lophomonas* spp. and *S. pyogenes* further underscores the unusual nature of the infection and its diagnostic complexity.

## Case presentation

A 28-year-old woman with a history of childhood asthma and a 10-year history of type 1 diabetes mellitus managed with insulin presented to the emergency department on December 26, 2024, due to severe epigastric pain radiating to the lumbar region, rated 10/10 on the visual analog scale, which improved following analgesic treatment. Her glycemic control had been suboptimal, with documented episodes of recurrent hyperglycemia. At presentation, her blood glucose level was 160 mg/dL, with persistent elevation throughout her hospital stay despite insulin optimization (Table 1).

**Table 1:** Serial blood glucose measurements during hospitalization.

Date	Glucose value (mg/dL)	Reference range (mg/dL)
26 Dec 2024	160	70–99
02 Jan 2025	130	70–99
04 Jan 2025	219	70–99
07 Jan 2025	259	70–99
08 Jan 2025	302	70–99
11 Jan 2025	165	70–99
15 Jan 2025	129	70–99
17 Jan 2025	208	70–99
19 Jan 2025	270	70–99
21 Jan 2025	141	70–99
23 Jan 2025	229	70–99
25 Jan 2025	221	70–99

**Figure 1:**
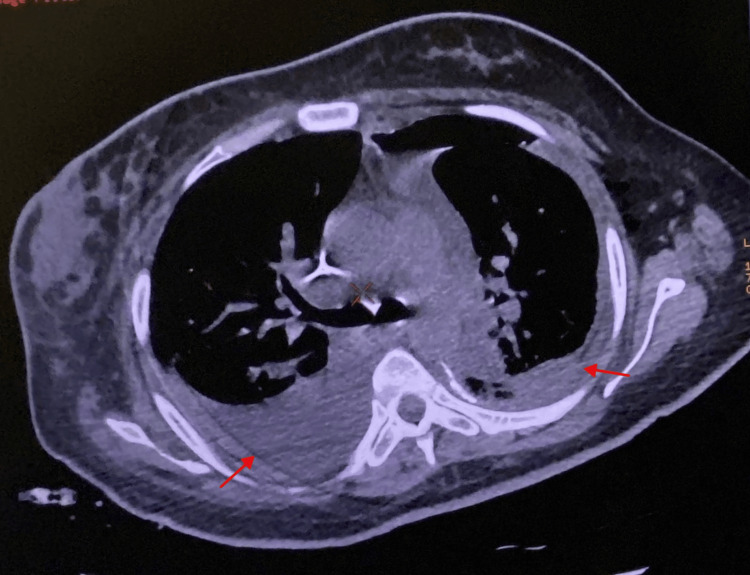
CT scan showing bilateral pleural effusions.

**Figure 2:**
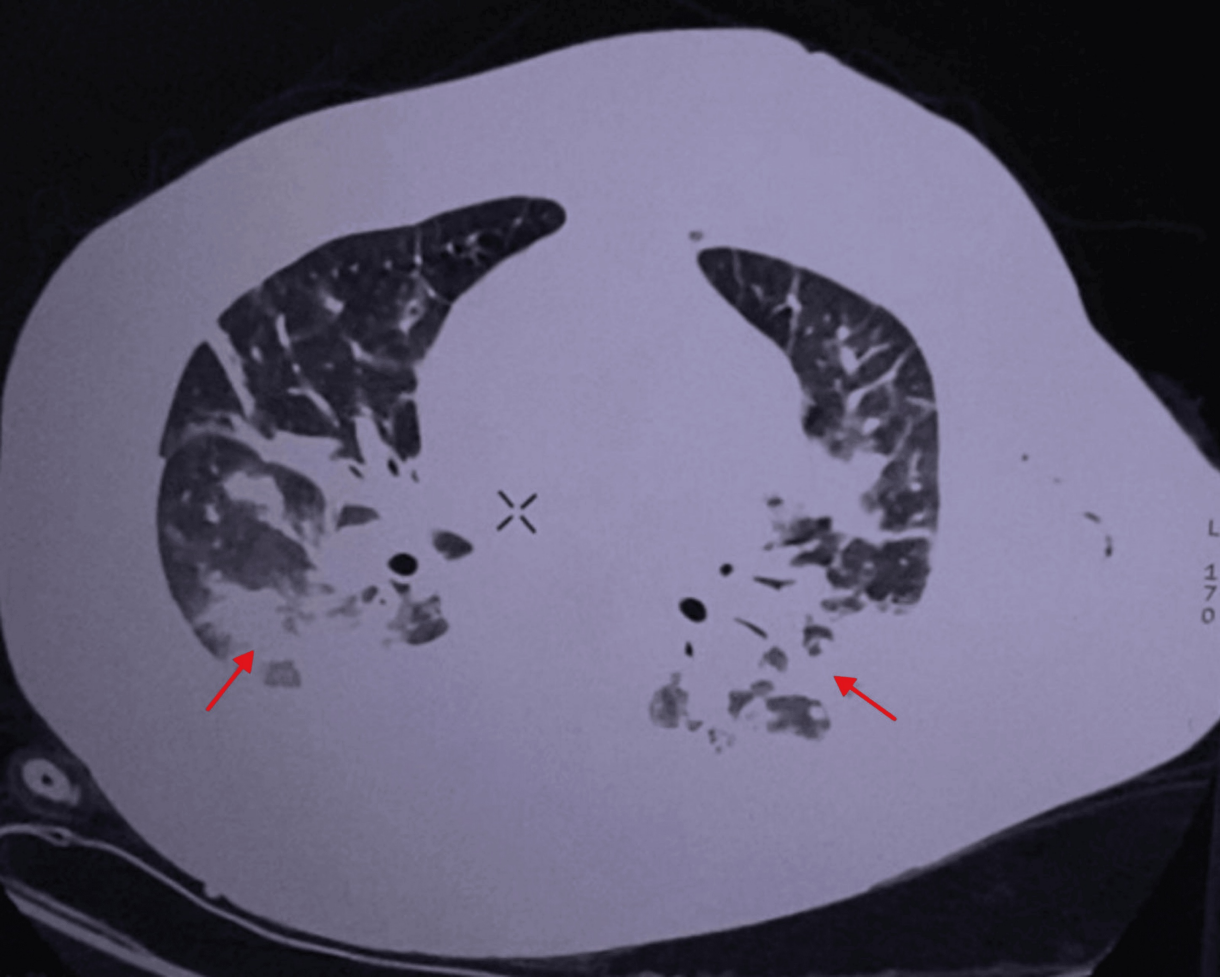
CT scan showing ground-glass opacities and subsegmental atelectasis.

On the second day of admission, arterial blood gas analysis findings were consistent with respiratory alkalosis secondary to hypoxemia, which improved following the administration of supplemental oxygen (Table 2). Chest CT demonstrated a significant left pleural effusion (~326 mL) and a smaller right pleural effusion (~30 mL) (Figure 1). The left lower lobe showed multiple confluent consolidations, intralobular interstitial thickening, and posterior ground-glass opacities. The right lower lobe showed subsegmental atelectasis and ground-glass opacities along the medial borders of the superior and posterior basal segments (Figure 2).

**Table 2:** ABG results on the second day of admission. ABG: Arterial blood gas

Parameter	Patient value	Reference range
pH	7.49	7.35–7.45
pO₂, mmHg	36	80–100
pCO₂, mmHg	30	35–45
HCO₃⁻, mmol/L	29.4	22–28
Base excess, mmol/L	6	-2 to +2
SaO₂, %	75	95–100

Given the lack of clinical improvement, empiric broad-spectrum antimicrobial therapy with meropenem and vancomycin was initiated on December 28, 2024; however, no clinical or radiological improvement was noted. A follow-up chest X-ray three days later showed increased left hemithorax opacity, prompting thoracentesis.

Pleural fluid analysis revealed a lymphocyte-predominant exudate with elevated protein concentration. Gram stain and acid-fast bacilli smears were negative, and initial bacterial and fungal cultures showed no growth. These findings raised suspicion for alternative etiologies, including autoimmune conditions or tuberculosis (TB). Serologic testing for anti-double-stranded DNA (anti-dsDNA) antibodies and measurement of adenosine deaminase levels in pleural fluid were negative for TB. HIV testing was non-reactive. Laboratory results demonstrated a C-reactive protein (CRP) level of 32.7 mg/L and a moderate leukocytosis of 11,400 cells/mm³.

**Figure 3:**
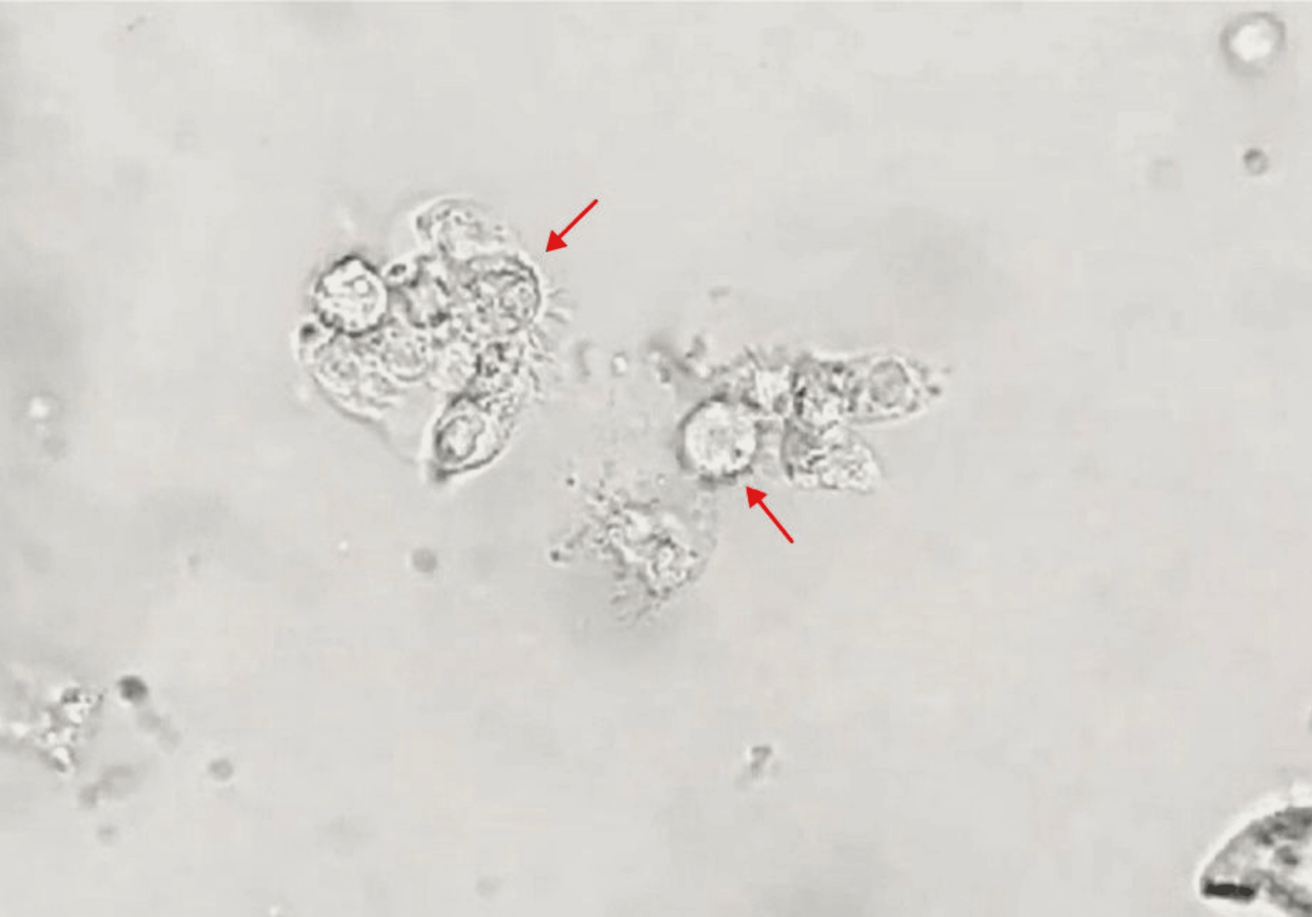
Direct microscopic sample demonstrating Lophomonas spp. from bronchial secretions.

Given the inconclusive results and persistent abnormalities, bronchoalveolar lavage (BAL) was performed on January 4, 2025. Potassium hydroxide (KOH) staining was negative for fungal elements. Subsequently, thoracoscopy with pleural and pulmonary biopsies revealed histopathological findings compatible with an inflammatory process of probable infectious origin. Flexible bronchoscopy with bronchial secretion aspiration identified motile, multiflagellated protozoa morphologically consistent with *Lophomonas* spp. (Figure 3; Video 1). Based on these findings, metronidazole therapy was initiated leading to progressive clinical improvement.

**Video 1:** Direct microscopic sample demonstrating Lophomonas spp.

Nevertheless, on January 14, 2025, the patient developed febrile episodes reaching 39 °C, accompanied by nausea. A repeat chest CT scan revealed new pulmonary infiltrates. A multiplex respiratory PCR (FilmArray) detected *S. pyogenes* as a potential secondary pathogen (Table 3). The antibiotic regimen was subsequently adjusted to benzylpenicillin.

**Table 3:** FilmArray respiratory panel demonstrating a positive result for S. pyogenes.

Sample: Bronchial lavage	
Bacteria	Result
Acinetobacter	Undetected
*Calcoaceticus-baumannii *complex	Undetected
*Enterobacter cloacae *complex	Undetected
Escherichia coli	Undetected
Haemophilus influenzae	Undetected
Klebsiella aerogenes	Undetected
Klebsiella oxytoca	Undetected
*Klebsiella pneumoniae *group	Undetected
Moraxella catarrhalis	Undetected
Proteus spp.	Undetected
Pseudomonas aeruginosa	Undetected
Serratia marcescens	Undetected
Staphylococcus aureus	Undetected
Staphylococcus agalactiae	Undetected
Streptococcus pneumoniae	Undetected
Streptococcus pyogenes	Detected
Atypical bacteria:	
Chlamydia pneumoniae	Undetected
Legionella pneumophila	Undetected
Mycoplasma pneumoniae	Undetected
Virus:	
Adenovirus	Undetected
Coronavirus	Undetected
Human metapneumovirus	Undetected
Rhinovirus/human enterovirus	Undetected
Influenza A	Undetected
Influenza B	Undetected
Parainfluenza virus	Undetected
Respiratory syncytial virus	Undetected

The patient remained hemodynamically stable and afebrile for the remainder of her hospital stay. On physical examination, residual subcrepitant rales were noted, with no additional significant findings. Laboratory parameters returned to normal limits. She was discharged on January 25, 2025, in a stable and satisfactory condition, with outpatient follow-up arranged.

## Discussion

Pulmonary lophomoniasis is an infrequent infection caused by *Lophomonas *spp., a flagellated protozoan first identified in China over thirty years ago. Since then, China has reported the highest number of cases, establishing itself as the region with the greatest prevalence worldwide [8]. In Mexico, only four cases have been documented: two initial cases in 2017 in the states of Hidalgo and Queretaro, a third case in 2019 in Tabasco, and the most recent one in 2022 in Oaxaca [9]. This low reported incidence suggests a possible underestimation of the disease, likely due to limited awareness of the parasite, diagnostic challenges, and potential confusion with other pulmonary pathogens.

Globally, infections caused by* Lophomonas* spp. continue to be considered rare. A systematic review indicated that aside from China, cases have also been reported in countries such as Peru and Spain [8]. The infection appears to affect male patients predominantly and has been documented in both immunocompromised and immunocompetent individuals. Furthermore, a higher prevalence has been observed among younger patients, suggesting that youth may represent a risk factor [10]. Poorly controlled type 1 diabetes mellitus is a well-recognized cause of functional immunosuppression. Chronic hyperglycemia impairs multiple components of the immune response, including neutrophil chemotaxis, phagocytosis, and intracellular killing. Additionally, it reduces cytokine production and compromises mucosal barrier defenses. These defects contribute to increased susceptibility to both opportunistic and conventional infections, including parasitic and bacterial pathogens [11]. In this case, poorly controlled type 1 diabetes mellitus created an immunologically vulnerable environment that favored infection by Lophomonas spp. and subsequent bacterial coinfection with S. pyogenes. Chronic hyperglycemia impairs neutrophil function, cytokine release, and epithelial barrier integrity, enabling persistence of both pathogens.

The clinical presentation of pulmonary lophomoniasis is nonspecific and commonly includes fever, cough, and dyspnea [12]. Laboratory findings may reveal peripheral eosinophilia, elevated CRP, and an increased erythrocyte sedimentation rate. Definitive diagnosis relies on identification of motile, multiflagellated trophozoites in sputum or BAL samples, which is critical for distinguishing *Lophomonas *spp. from other protozoa such as *Trichomonas* spp. or environmental ciliates. In this context, pulmonary infections caused by free-living amoebae like *Acanthamoeba* spp. and *Vermamoeba vermiformis* have also been reported and should be included in the differential diagnosis [13]. CT scans often reveal nonspecific findings, including consolidations, interstitial thickening, or ground-glass opacities [14]. The diagnosis in this patient was established through direct microscopy of bronchial secretions; however, no molecular confirmation was performed due to limited resources. This constitutes a limitation of the present report and underscores the need for molecular tools to improve diagnostic accuracy. 

Conversely, *S. pyogenes* is a versatile pathogen responsible for a wide spectrum of infections, ranging from pharyngitis to severe invasive diseases such as pneumonia, necrotizing fasciitis, and streptococcal toxic shock syndrome [15]. Although commonly associated with upper respiratory tract infections, *S. pyogenes* can also cause pneumonia, typically presenting in an interstitial pattern. Recent epidemiological data indicate an increasing incidence of invasive *S. pyogenes* infections, particularly in Europe, attributed to shifts in transmission dynamics and the virulence of specific strains [16].

In terms of treatment, pulmonary lophomoniasis responds favorably to metronidazole, administered orally or intravenously, with no documented resistance to date [17]. For *S. pyogenes* infections, penicillin remains the first-line therapy. In severe cases, third-generation cephalosporins may be used, and combination therapy with clindamycin is recommended in invasive infections due to its ability to inhibit toxin synthesis. However, clindamycin should not be used as monotherapy because its bacteriostatic action may promote resistance development [18-20].

## Conclusions

Pulmonary lophomoniasis constitutes an emerging diagnostic challenge in clinical practice due to its low prevalence, nonspecific symptomatology, and morphological similarity to other protozoa. This clinical case highlights the importance of including *Lophomonas* spp. in the differential diagnosis of atypical pneumonia, particularly in young patients with evident immunosuppression and poorly controlled diabetes with documented environmental exposure to vectors such as cockroaches. Moreover, the rare coinfection with *S. pyogenes* underscores the necessity for careful monitoring for new symptoms during the clinical course. 

The coexistence of *Lophomonas* spp. and *S. pyogenes* in this patient may reflect epithelial injury and immune dysfunction secondary to chronic hyperglycemia, emphasizing the interplay between protozoal and bacterial pathogens in immunocompromised hosts. Prompt identification of the etiological agent through direct microbiological methods, such as fresh examination of bronchial secretions, allowed for targeted therapeutic intervention, resulting in a favorable response to metronidazole and benzylpenicillin. This case exemplifies the value of conventional diagnostic techniques, a multidisciplinary clinical approach, and the consideration of unusual pathogens in diabetic patients presenting with atypical or refractory pneumonia. A limitation of this report is that molecular confirmation by PCR was not performed due to resource constraints, which highlights the need to expand molecular diagnostic availability in future studies.

## References

[1] Mewara A, Gile GH, Mathison B, Zhao H, Pritt B, Bradbury RS (2024). Lophomonas as a respiratory pathogen-jumping the gun. J Clin Microbiol.

[2] Nakhaei M, Fakhar M, Sharifpour A, Ziaei Hezarjaribi H, Banimostafavi ES, Nazar E (2022). Global status of emerging Lophomonas infection: a systematic review of reported cases (1993-2020). Interdiscip Perspect Infect Dis.

[3] Morales MG, Ceferino CY, Cadenas CJ, Méndez AG (2019). Pulmonary lophomoniasis (Article in Spanish). Med Crit.

[4] Kalani H, Pangh A, Nakhaei M (2022). High occurrence of emerged Lophomonas infection among patients suspected of having pulmonary tuberculosis: in-house PCR-based evidence. Interdiscip Perspect Infect Dis.

[5] Tąpolska-Jóźwiak K, Gowin E, Pasieka A (2024). Increasing incidence of severe complicated pneumonia in children caused by Streptococcus pyogenes. Pediatr Pulmonol.

[6] Enkel SL, Wong B, Hla TK (2024). Transmission potential of Streptococcus pyogenes during a controlled human infection trial of pharyngitis. mSphere.

[7] Al-Kaabi N, Solh Z, Pacheco S, Murray L, Gaboury I, Le Saux N (2006). A comparison of group A Streptococcus versus Streptococcus pneumoniae pneumonia. Pediatr Infect Dis J.

[8] Martinez-Girón R, Cornelis van Woerden H (2013). Lophomonas blattarum and bronchopulmonary disease. J Med Microbiol.

[9] Carrasco-Vargas H, Avendaño-Botello E, Carreón-Bonilla BP, Márquez-Jiménez A, Vargas-García ZD, Jaguey-Camarena KW (2024). Atypical pneumonia due to Lophomonas blattarum in an immunocompromised patient: a case report (Article in Spanish). Rev Sanid Milit.

[10] Ding Q, Shen K (2021). Pulmonary Infection with Lophomonas blattarum. Indian J Pediatr.

[11] Bilgic S, Aktas E, Salman F, Ersahin G, Erten G, Yilmaz MT, Deniz G (2008). Intracytoplasmic cytokine levels and neutrophil functions in early clinical stage of type 1 diabetes. Diabetes Res Clin Pract.

[12] Zhang X, Xu L, Wang LL, Liu S, Li J, Wang X (2011). Bronchopulmonary infection with Lophomonas blattarum: a case report and literature review. J Int Med Res.

[13] Nisar MA, Ross KE, Brown MH, Bentham R, Hinds J, Whiley H (2022). Molecular screening and characterization of Legionella pneumophila associated free-living amoebae in domestic and hospital water systems. Water Res.

[14] Wang Y, Tang Z, Ji S (2006). Pulmonary Lophomonas blattarum infection in patients with kidney allograft transplantation. Transpl Int.

[15] Brouwer S, Rivera-Hernandez T, Curren BF (2023). Pathogenesis, epidemiology and control of Group A Streptococcus infection. Nat Rev Microbiol.

[16] Rümke LW, Davies MA, Vestjens SM (2024). Nationwide upsurge in invasive disease in the context of longitudinal surveillance of carriage and invasive Streptococcus pyogenes 2009-2023, the Netherlands: a molecular epidemiological study. J Clin Microbiol.

[17] Löfmark S, Edlund C, Nord CE (2010). Metronidazole is still the drug of choice for treatment of anaerobic infections. Clin Infect Dis.

[18] van Driel ML, De Sutter AI, Habraken H, Thorning S, Christiaens T (2016). Different antibiotic treatments for group A streptococcal pharyngitis. Cochrane Database Syst Rev.

[19] Hedin K, Thorning S, van Driel ML (2023). Different antibiotic treatments for group A streptococcal pharyngitis. Cochrane Database Syst Rev.

[20] Silva-Costa C, Friães A, Ramirez M, Melo-Cristino J (2015). Macrolide-resistant Streptococcus pyogenes: prevalence and treatment strategies. Expert Rev Anti Infect Ther.

